# Nanoplastics and Immune Disruption: A Systematic Review of Exposure Routes, Mechanisms, and Health Implications

**DOI:** 10.3390/ijms26115228

**Published:** 2025-05-29

**Authors:** Dariusz Skaba, Jakub Fiegler-Rudol, Diana Dembicka-Mączka, Rafał Wiench

**Affiliations:** 1 Department of Periodontal Diseases and Oral Mucosa Diseases, Faculty of Medical Sciences in Zabrze, Medical University of Silesia, 40-055 Katowice, Poland; s88998@365.sum.edu.pl (J.F.-R.); rwiench@sum.edu.pl (R.W.); 2 Dental Office—Artistic Smile Studio, 61/1 Krakowska Street, 33-100 Tarnów, Poland; dianadembicka@vp.pl

**Keywords:** nanoplastics, inflammation, cytokines, oxidative, toxicity, autoimmunity, health

## Abstract

Plastic waste degradation has led to an increase in nanoplastics, which can cross biological barriers and disrupt immune function, potentially triggering inflammatory and autoimmune conditions. A systematic review was conducted following PRISMA 2020 guidelines. Literature from PubMed, Embase, Scopus, and Cochrane Library (2015–2025) was screened for in vitro and in vivo studies investigating nanoplastic-induced immune responses, with data extraction and quality assessment performed by independent reviewers. Ten studies met inclusion criteria. Both in vitro and in vivo evidence demonstrated that nanoplastic exposure induces oxidative stress, cytokine imbalance, and activation of pro-inflammatory pathways, resulting in tissue-specific cellular damage across multiple organs. Nanoplastic exposure is linked to significant immune modulation and inflammation, indicating potential public health risks. Further long-term, standardized research is needed to elucidate the role of nanoplastics in autoimmune diseases such as lichen planus and to inform regulatory policies.

## 1. Introduction

### 1.1. Rationale

Plastic production has increased exponentially over the past several decades, leading to the accumulation of vast amounts of plastic waste in the environment, which in turn has accelerated the degradation of larger plastic items into micro- and nanoplastics [1,2,3,4,5,6]. Nanoplastics, defined as particles typically 1–100 nm in size, are generated either through the weathering of macroplastics or are directly manufactured for specialized applications, thereby contributing to an ever-growing presence in diverse environmental compartments [7,8,9,10]. Micro- and nanoplastics can infiltrate the food chain through multiple routes, ultimately leading to dietary exposure in consumers. Their presence has been identified in various food items, including honey, table salt, milk, mineral water, and seafood, offering evidence of human ingestion through contaminated food sources [7,11,12,13,14]. Due to their exceptionally small size and large surface area-to-volume ratio, nanoplastics exhibit unique physicochemical properties that not only distinguish them from their bulk counterparts but also enhance their potential to interact with biological systems [15,16,17,18,19,20]. These interactions are particularly concerning given that nanoplastics can easily traverse biological barriers, such as the gastrointestinal lining, pulmonary epithelium, and even the dermal layers, ultimately entering systemic circulation [21,22,23]. While both microplastics and nanoplastics are relevant to human exposure, nanoplastics are of particular concern due to their smaller size and distinct physicochemical properties, which enhance their ability to penetrate biological barriers and interact with cellular and molecular systems [15,16,17,18,19,20]. These properties differentiate nanoplastics from their larger counterparts and are the primary rationale for focusing this review specifically on nanoplastics.

Once in the bloodstream, nanoplastics have been shown to interact with a variety of blood components, including plasma proteins and hematopoietic cells, thereby raising significant concerns regarding their potential to disrupt immune homeostasis [24]. Moreover, these minute particles can adsorb various environmental co-pollutants—such as heavy metals, pesticides, and persistent organic pollutants—further amplifying their toxicological profile and complicating their biological interactions [25,26]. The combined presence of nanoplastics and these co-pollutants may lead to a cascade of adverse cellular responses, including the induction of oxidative stress, DNA damage, and subsequent inflammatory reactions, which are critical factors in the modulation of immune function [27,28,29]. Emerging in vitro and in vivo studies have highlighted that nanoplastic exposure can activate pro-inflammatory signaling pathways, alter cytokine production, and modulate the activities of key immune cells, thereby suggesting a link between nanoplastic contamination and immune dysregulation [29,30]. These immunomodulatory effects are particularly significant because a properly functioning immune system is essential not only for defending the host against pathogens but also for maintaining overall physiological balance and preventing chronic diseases [31]. Understanding the mechanistic underpinnings of how nanoplastics modulate immune responses is thus of paramount importance, as such insights could pave the way for developing strategies to mitigate their adverse health effects [32,33]. In recent years, research has increasingly focused on characterizing the interaction between nanoplastics and blood components, with studies indicating that these interactions can lead to alterations in protein conformation and function, thereby compromising the blood’s ability to transport essential molecules and mount effective immune responses [34]. Furthermore, nanoplastics have been implicated in triggering hemolytic events, altering red blood cell morphology, and even inducing the formation of protein coronas that may obscure the particle’s identity and influence its biodistribution and toxicity [35]. The formation of these protein coronas is a dynamic process that not only affects the physicochemical characteristics of the nanoplastics but also determines their subsequent cellular uptake and immunological fate, potentially leading to enhanced cytotoxicity and immune activation [36,37]. Given the ubiquity of nanoplastics in the environment and their ability to enter the human body through multiple exposure routes—such as ingestion, inhalation, and dermal contact—the potential health implications are broad and multifaceted [38]. Chronic exposure to nanoplastics may result in a sustained inflammatory state that can predispose individuals to a range of diseases, including autoimmune disorders, cardiovascular ailments, and even neurodegenerative conditions [20,39,40]. Despite the growing body of evidence, many uncertainties remain regarding the precise mechanisms by which nanoplastics exert their immunotoxic effects, particularly under real-life exposure scenarios where multiple stressors and co-pollutants are present [41,42]. To address these critical gaps in knowledge, this systematic review aimed to consolidate and critically evaluate the current evidence on the immunomodulatory effects of nanoplastic exposure, with a focus on elucidating the underlying molecular pathways and assessing the resultant health outcomes [42,43,44,45].

### 1.2. Objectives

This systematic review aimed to systematically evaluate the available literature on the interactions between nanoplastics and the immune system by synthesizing evidence from both in vitro and in vivo studies. Its objectives included elucidating the mechanisms by which nanoplastics influence cytokine secretion, inflammatory cell activation, and immune regulatory networks, assessing their potential role in the development of chronic inflammatory conditions, identifying existing knowledge gaps, and ultimately providing a comprehensive framework to inform future research, risk assessment, and regulatory policies regarding nanoplastic exposure and its public health implications.

## 2. Materials and Methods

### 2.1. Focused Question

This systematic review was conducted following the PICO framework [46] to examine the impact of nanoplastic exposure on immune system function and related health outcomes. The research question was structured as follows: Among individuals exposed to nanoplastics (population), does nanoplastic exposure (intervention) lead to significant immune modulation or adverse health effects (outcome) compared to individuals with minimal or no exposure (comparison)?

### 2.2. Search Strategy

This systematic review, registered with PROSPERO (CRD420250651925), followed PRISMA 2020 guidelines. A comprehensive search across PubMed, Embase, Scopus, and the Cochrane Library (2015–2025, English, full-text) used standardized MeSH terms related to nanoplastics and immune responses. Three independent researchers conducted the search and screening, with full-text reviews by two reviewers and additional studies identified via snowballing. The review aimed to assess the impact of nanoplastic exposure on immune system modulation and related health outcomes, based on predefined inclusion and exclusion criteria.

### 2.3. Selection of Studies

To maintain objectivity in the selection process, the reviewers independently evaluated the titles and abstracts of all retrieved studies. Any disagreements concerning study eligibility were addressed through in-depth discussions until a consensus was achieved. This systematic approach, aligned with PRISMA guidelines, aimed to strengthen the review’s methodological integrity by including only the most relevant and scientifically robust studies [47]. Studies were included if they investigated the effects of nanoplastic exposure on immune system function in humans or animal models, specifically examining immune responses such as inflammation, cytokine production, and immune cell activity. Eligible studies assessed health outcomes related to immune modulation, including autoimmune diseases, increased susceptibility to infections, or inflammatory disorders. Both experimental (in vitro, in vivo) and epidemiological studies analyzing nanoplastic accumulation and its impact on immune homeostasis were considered, as well as comparative analyses evaluating immune responses in nanoplastic-exposed versus non-exposed populations. Longitudinal studies or those with follow-up periods to assess sustained immune effects were also included, provided they employed well-defined methodologies to quantify nanoplastic exposure and its immunological consequences. Only peer-reviewed studies published in English were eligible. Exclusion criteria encompassed non-peer-reviewed sources, studies published in languages other than English, duplicate studies or those sharing the same ethical approval number, and gray literature such as case reports, letters to editors, narrative or systematic reviews, books, conference abstracts, and non-journal materials. Additionally, studies that did not specifically investigate immune modulation in relation to nanoplastic exposure, focused solely on toxicokinetics or environmental fate without immune assessment, in vitro studies lacking analysis of immune cell responses or pathways, research involving only microplastics, or studies addressing general environmental pollution without isolating nanoplastics as a variable were excluded.

### 2.4. Risk of Bias in Individual Studies

During the preliminary stage of study selection, reviewers independently examined the titles and abstracts of identified studies to minimize bias in the screening process. To assess the reliability of their evaluations, Cohen’s kappa statistic was utilized to quantify inter-reviewer agreement [48]. Any disagreements concerning the inclusion or exclusion of studies were addressed through thorough discussions among the authors until a unanimous decision was achieved.

The methodological quality of the included studies was independently assessed by three authors, focusing on critical aspects of nanoplastic exposure assessment, study design, and the reporting of immune-related outcomes to ensure objectivity and validity. Risk of bias was evaluated using a structured checklist, assigning a score of 1 for a “yes” response and 0 for a “no” response to the following criteria: (1) clear definition of the size, shape, and composition of the nanoplastics; (2) explicit reporting of exposure concentration or dose; (3) clear description of the exposure method (e.g., ingestion, inhalation, dermal); (4) inclusion of appropriate control groups (e.g., vehicle controls, untreated groups); (5) provision of details on nanoplastic preparation and administration to support reproducibility; (6) use of blinding or randomization to reduce selection and detection bias; (7) reporting of immune-related endpoints (e.g., cytokine levels, immune cell activation, inflammatory markers) with statistical analysis; (8) absence of missing or selectively reported outcome data; and (9) lack of conflicts of interest related to funding sources. Based on the number of “yes” responses, each study was categorized as having a high (0–3), moderate (4–6), or low (7–9) risk of bias. The overall risk of bias was determined according to the guidelines of the *Cochrane Handbook for Systematic Reviews of Interventions* [49], ensuring a standardized and rigorous assessment of study reliability and validity. Table 1 presents the results of the risk of bias assessment across studies.

### 2.5. Data Extraction

Once an agreement was reached on the selected articles for inclusion, both authors systematically extracted relevant information. Both authors independently utilized a standardized data extraction form to ensure consistency and minimize bias. Key information was meticulously retrieved from each selected article, including citation details (first author and publication year), study design, and specifics of nanoplastic exposure (such as type, concentration, and route). In addition, details on the immune modulation outcomes, ranging from cytokine responses and inflammatory markers to cellular alterations, were carefully recorded. Other essential parameters, such as the composition of test and control groups, follow-up duration, and any relevant methodological details, were also captured. This comprehensive extraction process was fundamental in enabling a thorough synthesis of the available evidence and in enhancing the reliability and reproducibility of the review’s findings.

## 3. Results

### 3.1. Study Selection

**Figure 1:**
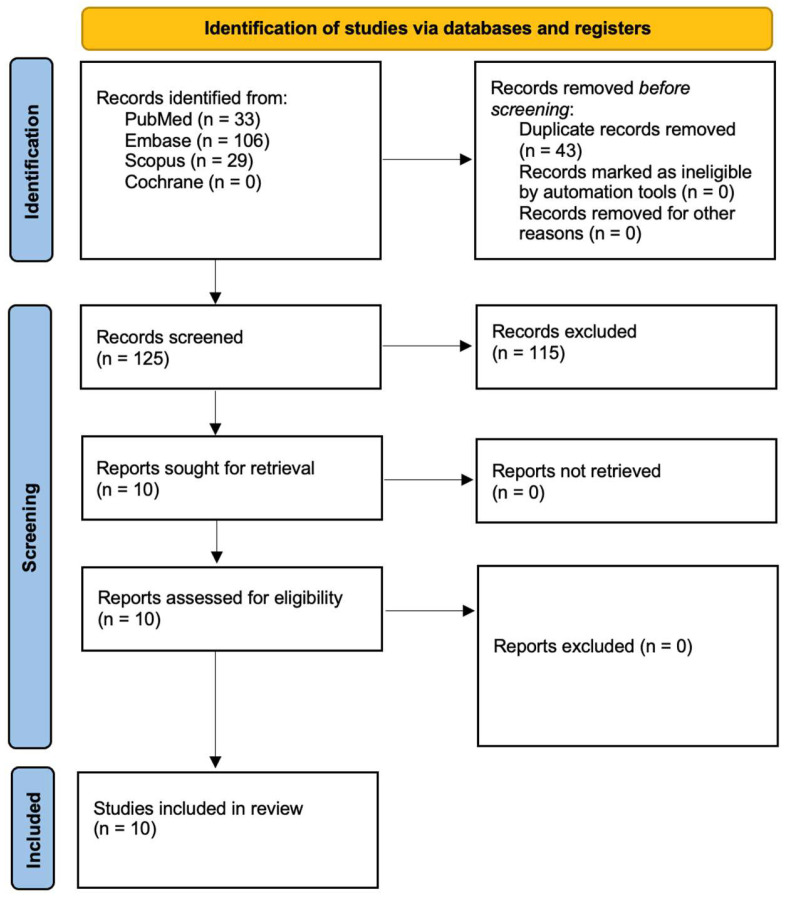
Prisma 2020 flow diagram.

In accordance with PRISMA guidelines [47], Figure 1 outlines the process used to select studies. The initial search produced 168 articles, which were reduced to 125 unique records after duplicates were removed. A detailed review of titles and abstracts led to the identification of 10 articles for full-text evaluation, with all being deemed relevant. Ultimately, these 10 studies, all published within the past decade, were incorporated into the final analysis.

### 3.2. Data Presentation

Table 2, Table 3 and Table 4 offer a summary of the essential data extracted from the 8 studies that satisfied the inclusion criteria and were ultimately incorporated into the review.

### 3.3. General Characteristics of the Included Studies

Table 2 provides an overview of the key features of the 10 studies that were incorporated into this review.

### 3.4. Main Study Outcomes

Busch et al. [50] demonstrated that in the intestine, nanoplastics have negligible acute effects under healthy conditions but, in the presence of inflammation, they induce a loss of epithelial cells, increased IL-1β release, DNA damage, and impaired barrier integrity, suggesting that pre-existing intestinal inflammation amplifies the immune response to nanoplastic exposure. Huang et al. [51] reported that in the esophagus, PVC nanoplastics trigger oxidative stress and inhibit homology-directed repair by downregulating key DNA repair genes, which activates the cGAS-STING signaling pathway and promotes the release of inflammatory cytokines such as TNF-α, IL-6, CCL5, CXCL10, and IFN-γ, ultimately leading to increased cellular senescence that can be mitigated by vitamin C. Lu et al. [52] found that in the kidneys, nanoplastics are internalized in a time- and dose-dependent manner, causing oxidative stress through mitochondrial damage, reactive oxygen species accumulation, increased lipid peroxidation, and suppression of antioxidant defenses, which together promote an inflammatory response and cellular senescence marked by elevated p16, p53, and H2AX levels alongside decreased mitochondrial membrane potential. Poinsignon et al. [53] observed that in the placenta, exposure to smaller 20 nm PS nanoplastics leads to rapid internalization by trophoblastic cells, resulting in higher ROS production, cytotoxicity, and an inflammatory response via NF-κB activation, as well as endocrine disruption evidenced by a dose-dependent reduction in β-hCG secretion. Weber et al. [54] showed that irregular PVC nanoplastics provoke a strong inflammatory response in immune cells, notably increasing the secretion of IL-6, TNF-α, and IL-10, whereas irregular PS nanoplastics elicit a milder response and spherical PS nanoplastics have minimal effects, indicating that both polymer type and particle shape are critical factors in modulating immune cell activity. In a similar study, Weber et al. [55] further confirmed that the inflammatory effects of nanoplastics are primarily driven by their physical presence rather than chemical leaching, underscoring the role of particle morphology in immune system activation. Ge et al. [56] reported that in the liver, chronic inhalation exposure to PS nanoplastics leads to ferroptosis-mediated liver injury and fibrosis characterized by increased markers of liver dysfunction, structural damage, heightened inflammatory responses, and biochemical evidence of oxidative stress, iron overload, and mitochondrial dysfunction, which could be mitigated by a ferroptosis inhibitor. Finally, Liu et al. [57] demonstrated that in the lungs, nasal instillation of PS nanoplastics induces pulmonary fibrosis through mitochondrial DNA release that activates the cGAS-STING pathway, leading to oxidative stress, apoptosis, necrosis, and an increase in pro-inflammatory cytokines, with inhibition of STING significantly reducing lung damage. Li et al. (2020) demonstrated that polystyrene nanoplastics (PS-NPs) accumulate in multiple organs of Corbicula fluminea, causing oxidative stress, inflammation, liver damage, and neurotoxicity, with the visceral mass being the most sensitive to immune-related toxicity [58]. Cid-Samamed et al. (2024) found that PS-NPs induced oxidative stress and disrupted antioxidant enzymes in Mytilus galloprovincialis, particularly affecting gills more than digestive glands, with toxicity influenced by exposure duration, dose, and aggregation methods [59].

## 4. Discussion

### 4.1. Results in the Context of Other Evidence

Nanoplastics, owing to their small size and large surface area, can easily cross biological barriers and disrupt immune homeostasis [50,51,52,53,54,55,56,57]. In inflamed intestinal environments, PVC nanoplastics notably increase the release of IL-1β and lead to epithelial cell loss, indicating an amplified toxicity under inflammatory conditions [50]. PVC nanoplastics also induce oxidative stress in esophageal cells by suppressing DNA repair mechanisms, activating the cGAS-STING pathway and thereby promoting inflammation and cellular senescence [51]. In kidney cells, nanoplastics are internalized in a time- and dose-dependent manner, triggering oxidative stress, mitochondrial damage, and an inflammatory cytokine surge that culminates in cellular aging [52]. Exposure to polystyrene nanoplastics in placental cells causes cytotoxicity, oxidative stress, and endocrine disruption by reducing hCG secretion, which may jeopardize placental health and fetal development [53]. The inflammatory response elicited by nanoplastics is significantly influenced by their polymer type and particle shape, with irregular fragments provoking a more pronounced cytokine release than their spherical counterparts [54]. In vivo studies reveal that long-term inhalation of PS nanoplastics results in liver injury and fibrosis via ferroptosis, underpinned by oxidative stress, iron overload, and heightened inflammatory markers [56]. Nasal instillation of PS nanoplastics in mice has been shown to induce pulmonary fibrosis through mitochondrial DNA release and subsequent cGAS-STING pathway activation, aggravating lung inflammation [57]. The collective evidence suggests that chronic nanoplastic exposure may foster a persistent inflammatory state, thereby predisposing individuals to autoimmune, cardiovascular, and neurodegenerative disorders [50,51,52,53,54,55,56,57]. Finally, the review underscores significant heterogeneity among studies and calls for standardized research protocols to more precisely define the immunotoxic mechanisms of nanoplastics and guide future regulatory policies [50,51,52,53,54,55,56,57].

Research on the immunotoxic effects of microplastics and nanoplastics reveals a multifaceted and evolving field with significant gaps in our understanding that warrant further investigation. Dan et al. [60] have demonstrated that, although the presence of MPs and NPs is well established in both marine organisms and the human body, the immunological impacts of these particles vary considerably depending on material properties such as size, shape, and composition, which in turn necessitates further exploration and the development of potential removal strategies that may involve physicochemical, microbial, or biological methods. Similarly, Ali et al. [61] underscore that micro- and nanoplastics (MNPs) are associated with a range of adverse health outcomes, including oxidative stress, inflammation, immune dysfunction, metabolic disruptions, and even potential carcinogenicity, while also highlighting that current research, particularly involving human subjects, remains insufficient and calls for future studies that address realistic exposure levels, dose-dependent effects, individual susceptibility, and a broader spectrum of plastic particle types. In addition, Yang et al. [62] emphasize that MPs can disrupt immune homeostasis by inducing oxidative stress, triggering inflammatory responses, and interacting with other environmental pollutants to enhance toxicity, which collectively highlight the urgent need for comprehensive research focused on the underlying mechanisms and the full spectrum of exposure risks in humans. Ingestion studies conducted in both fish and mice [63,64,65] have revealed that MPs can activate the immune system and disturb the delicate balance of gut microbiota, while further research [66,67,68,69] has shown that these particles can infiltrate critical organs, such as the liver, kidneys, brain, and bloodstream, potentially provoking innate immune responses that involve key immune cells like phagocytic leukocytes [70]. This immunotoxicity appears to be primarily driven by oxidative stress, as evidenced by the production of ROS and the release of danger-associated molecular patterns [71]. Furthermore, Xu et al. [72,73,74,75,76,77,78] point out that while most existing studies indicate that MNPs are linked to significant health risks, including cytotoxicity, oxidative stress, inflammation, genotoxicity, and organ toxicity, the research is often marred by inconsistencies in study methodologies and the lack of standardized benchmarks, which in turn highlights the need for more comprehensive and reproducible investigations. Finally, Jayavel et al. [79,80,81,82,83] bring attention to the fact that the environmental accumulation of microplastics in various ecosystems and food sources poses systemic health risks by affecting multiple organ systems and even causing genetic abnormalities, thereby emphasizing the importance of interdisciplinary research, the development of sustainable solutions, and improved waste management practices to mitigate these impacts.

### 4.2. Limitations of the Evidence

The evidence is constrained by significant variability in study design and methodology, making it challenging to draw definitive conclusions. Many studies employed in vitro models that may not fully replicate the complexity of human biological systems or real-world exposure scenarios, limiting the applicability of the findings. There is a lack of standardized protocols regarding nanoplastic characterization, exposure conditions, and measurement of immune responses, which leads to inconsistent and sometimes incomparable data. The predominance of short-term experiments further restricts understanding of the long-term health implications of nanoplastic exposure. Additionally, the exclusion of non-English literature and gray literature may have introduced selection bias, potentially omitting important findings. These limitations underscore the need for more comprehensive, standardized, and longitudinal research to better elucidate the immunotoxic effects of nanoplastics and their broader public health impact.

### 4.3. Limitations of the Review Process

This systematic review is limited by significant heterogeneity among the included studies, as variations in methodologies, exposure protocols, and outcome measures complicate direct comparisons and synthesis of results. The exclusion of non-English language publications and gray literature may have introduced publication bias, potentially omitting relevant data that could have influenced the overall conclusions. Moreover, the dominance of in vitro studies, alongside a relative scarcity of in vivo research, restricts the ability to generalize these findings to real-world human health scenarios. Inconsistencies in the reporting of nanoplastic characteristics and exposure conditions further hinder the establishment of standardized protocols, underscoring the need for more rigorous and unified research methodologies in future investigations. Additionally, the limitations inherent in the search strategy must be acknowledged. The choice of search terms—while carefully selected—inevitably constrains the scope of retrieved literature. For instance, the absence of broader or alternative keywords may have led to the omission of relevant studies that could have contributed important insights.

### 4.4. Implications for Practice, Policy, and Future Research

The implications of these findings extend across clinical practice, public health policy, and future scientific inquiry. In clinical practice, healthcare providers should consider environmental exposure to nanoplastics as a potential risk factor when diagnosing and managing autoimmune conditions such as lichen planus, as well as other chronic inflammatory disorders. This calls for a more integrative approach that includes environmental history in patient assessments, especially in regions with high levels of plastic pollution. For policymakers, these results underscore the urgent need for stricter environmental regulations regarding plastic production, disposal, and recycling, alongside comprehensive monitoring of nanoplastic levels in key ecosystems. Enhanced public awareness campaigns about the potential health risks associated with nanoplastic exposure may also promote safer practices among industries and consumers alike. Future research must focus on conducting long-term, standardized in vivo studies to elucidate the causative links between nanoplastic exposure and autoimmune diseases, determining critical exposure thresholds, and exploring potential therapeutic interventions. Such studies could pave the way for innovative prevention strategies and targeted treatments, ultimately contributing to a reduction in the incidence of environmentally induced immune disorders. As periodontologists, we believe that the potential impact of nanoplastics on oral potentially malignant disorders warrants further investigation, particularly in relation to oral lichen planus and its autoimmune etiology.

## 5. Conclusions

This systematic review underscores that nanoplastic exposure is linked to significant immune modulation, with both in vitro and in vivo studies indicating that these particles can induce oxidative stress, disrupt cytokine balance, and trigger inflammatory responses that may ultimately contribute to chronic inflammatory and autoimmune conditions. While the current body of evidence is limited by methodological heterogeneity and short-term experimental designs, the consistent demonstration of immune perturbations highlights the potential public health risks associated with environmental nanoplastic contamination. These findings emphasize the urgent need for standardized, long-term research to establish critical exposure thresholds and to investigate the etiological role of nanoplastics in autoimmune diseases, such as lichen planus, and other immune-mediated disorders. Ultimately, a deeper understanding of these mechanisms is essential to inform clinical practice, shape effective regulatory policies, and guide future interventions aimed at mitigating the adverse health outcomes of nanoplastic exposure.

## Figures and Tables

**Table 1:** The results of the quality assessment and risk of bias across the studies.

Study	1	2	3	4	5	6	7	8	9	Total	Risk
Busch et al. 2021 [50]	1	1	1	1	1	0	1	1	1	8	Low
Huang et al. 2025 [51]	1	1	1	1	1	0	1	1	1	8	Low
Lu et al. 2024 [52]	1	1	1	1	1	0	1	1	1	8	Low
Poinsignon et al. 2025 [53]	1	1	1	1	1	0	1	1	1	8	Low
Weber et al. 2022 [54]	1	1	1	1	1	0	1	1	1	8	Low
Wen et al. 2024 [55]	1	1	1	1	1	0	1	1	1	8	Low
Ge et al. 2024 [56]	1	1	1	1	1	0	1	1	1	8	Low
Liu et al. 2024 [57]	1	1	1	1	1	0	1	1	1	8	Low
Li et al. 2020 [58]	1	1	1	1	1	0	1	0	1	7	Low
Cid-Samamed et al. 2024 [59]	1	1	1	1	1	0	1	1	1	8	Low

**Table 2:** A general overview of the studies.

Author and Year	Country	Study Design
Busch et al. 2021 [50]	Germany	In vitro study
Huang et al. 2025 [51]	China and USA	In vitro study
Lu et al. 2024 [52]	China	In vitro study
Poinsignon et al. 2025 [53]	France	In vitro study
Weber et al. 2022 [54]	Germany and Norway	In vitro study
Wen et al. 2024 [55]	China	In vivo (mice) and in vitro (hepatocytes) study
Ge et al. 2024 [56]	China	In vivo (mice) study
Liu et al. 2024 [57]	China	In vivo (mice) study
Li et al. 2020 [58]	China	In vivo study
Cid-Samamed et al. 2024 [59]	Spain and Portugal	Proof-of-concept experimental study

**Table 3:** Main outcomes from the analyzed studies.

Author and Year	Target Organ/System	Mechanisms of Damage	Main Findings
Busch et al. 2021 [50]	Intestine	Loss of epithelial cells, increased IL-1β release, DNA damage, impaired barrier integrity (especially under inflammatory conditions)	- PVC nanoplastics induced immune and epithelial responses only in inflamed intestinal models. - PVC doubled IL-1β release and caused epithelial cell loss without necrosis in inflamed cultures. - PS and PS–NH₂ had no effect in triple cultures; PS–NH₂ increased IL-1β in THP-1 monocultures. - No cytotoxicity or DNA damage from PS or PVC in healthy models. - Inflamed intestines are more susceptible to nanoplastic-induced immune activation and cell loss, particularly from PVC.
Huang et al. 2025 [51]	Esophagus	Oxidative stress, inhibition of DNA repair (homology-directed repair suppression), cGAS-STING activation, inflammation, and cellular senescence	- PVC nanoplastics impair homology-directed repair by downregulating BRCA2 and GRB2, causing genomic instability. - Exposure leads to cell cycle arrest and elevated senescence markers (p16, p21, IL-6, TNF-α). - DNA damage triggers cGAS-STING activation, with TBK1/IRF3 phosphorylation and increased IFN-γ, TNF-α, IL-6, CCL5, CXCL10. - In mice, PVC caused esophageal inflammation, senescence, and behavioral changes (reduced activity, increased grooming).
Lu et al. 2024 [52]	Kidneys	Oxidative stress, mitochondrial damage, ROS accumulation, inflammation, and cellular senescence	- Nanoplastics increased pro-inflammatory cytokines (TNF-α, IL-6, IL-1β) in porcine kidney cells, indicating immune activation. - Exposure raised ROS and MDA levels, reduced SOD and TAC, and disrupted redox balance. - Senescence markers (p16, p53, H2AX) increased via oxidative stress, reversible by antioxidants (MnTBAP, TEMPO). - Decreased mitochondrial membrane potential and increased LDH release indicated impaired metabolism and enhanced inflammation.
Poinsignon et al. 2025 [53]	Placenta	Cytotoxicity, inflammatory response (IL-1β, IL-6, TNF-α), endocrine disruption (reduced β-hCG hormone levels)	- PS-NPs triggered pro-inflammatory responses in placental trophoblasts via NF-κB, with PS-NP20 more potent at high doses and PS-NP100 inducing IL-1β even at low doses. - Exposure caused ROS generation, especially with PS-NP20, and activated the Nrf2/HO-1 pathway with increased SOD and catalase. - PS-NP100 accumulated in lysosomes, initiated autophagy (Beclin-1, P62), but blocked autophagic flux, indicating impaired clearance and possible lysosomal damage. - Both PS-NP sizes reduced β-hCG secretion; PS-NP100 did so dose-dependently, suggesting direct disruption of hormone production.
Weber et al. 2022 [54]	Immune system (monocytes, dendritic cells)	Inflammatory cytokine release from immune cells (IL-6, TNF-α, IL-12p70, IL-23), immune system activation, polymer and shape-dependent toxicity	- Irregular PVC nanoplastics strongly induced IL-6, TNF, and IL-10 secretion in human monocytes, indicating potent immune activation. - Irregular shapes, especially PVC, triggered stronger responses than spherical particles, showing shape and polymer composition influence immunogenicity. - Minimal cytokine release from leachates confirmed physical properties, not chemicals, as primary immune triggers. - Responses were dose-dependent and varied by donor, indicating inter-individual susceptibility.
Wen et al. 2024 [55]	Liver	Polystyrene nanoplastics caused liver damage via ROS generation, NRF2 suppression, and NF-κB/NLRP3-mediated inflammation	- PS-NPs induced excess ROS and suppressed NRF2 signaling, causing oxidative liver damage in mice and AML-12 cells. - Inflammation was activated via the NF-κB/NLRP3 pathway, with increased NF-κB, IL-6, IL-1β, NLRP3, and caspase-1 expression. - NRF2 activation (tBHQ) reduced oxidative stress and inflammation, while inhibition (brusatol) reversed these effects. - Histopathology and elevated AST/ALT confirmed liver injury driven by oxidative stress and immune activation.
Ge et al. 2024 [56]	Liver	Polystyrene nanoplastics caused liver injury and fibrosis by inducing oxidative stress and iron overload, which trigger ferroptosis, a form of regulated cell death	- Inhaled PS-NPs caused dose- and time-dependent liver damage, elevating cytokines (IL-6, TNF-α, MCP-1) and liver enzymes (ALT, AST, ALP). - Long-term exposure led to liver fibrosis, confirmed by Masson staining and increased TGF-β, α-SMA, and Col1a1 expression. - PS-NPs triggered oxidative stress, lipid peroxidation, iron overload, and ferroptosis (↓GPX4, ↑TFRC, ↓Ferritin) in vivo and in vitro. - Ferroptosis inhibitor Fer-1 reduced liver injury, inflammation, and fibrosis, confirming ferroptosis as a key mechanism of PS-NP hepatotoxicity.
Liu et al. 2024 [57]	Lungs	Polystyrene nanoplastics caused lung injury by inducing mitochondrial DNA release and activating the cGAS-STING signaling pathway, which promotes inflammation, apoptosis, and pulmonary fibrosis	- PS-NPs activated the cGAS-STING pathway in RAW264.7 cells and mouse lungs, increasing cGAS, STING, TBK1, IRF3, NF-κB, and proinflammatory cytokines (IL-6, IL-1β). - Mitochondrial damage led to mtDNA leakage into cytosol, serum, and BALF, triggering immune activation. - Nasal exposure caused lung inflammation, macrophage recruitment, fibrosis, and elevated markers (α-SMA, Col1a1) in mice. - STING inhibitor C176 reduced lung injury and inflammation, confirming cGAS-STING’s key role in PS-NP-induced immune toxicity.
Li et al. 2020 [58]	Gill, intestine, stomach, liver, mantle, and visceral mass	Polystyrene nanoplastics induced oxidative stress by generating excess ROS, disrupting antioxidant enzymes, and causing lipid peroxidation, liver damage, intestinal inflammation, and neurotoxicity	- Polystyrene nanoplastics accumulated in multiple organs of Corbicula fluminea via ingestion, adherence, and respiration. - Exposure increased ROS and disrupted antioxidant enzymes (↑SOD, CAT, GPx; ↓GR, GSH), especially in the visceral mass. - PS-NPs caused intestinal inflammation (↑DAO, D-lactate), liver damage (↑GPT, GOT), and neurotoxicity (↓AChE), indicating systemic immune toxicity. - Effects were dose-dependent, with the visceral mass showing the strongest biomarker response.
Cid-Samamed et al. 2024 [59]	Gills and digestive glands	Polystyrene nanoplastics induced oxidative stress and antioxidant disruption in mussels by increasing reactive oxygen species (ROS), altering enzyme activities (SOD, CAT, GST, GPx), and enhancing lipid peroxidation	- PS-NH₂ NPs induced oxidative stress in Mytilus galloprovincialis, shown by changes in SOD, CAT, GST, GPx activities, and increased MDA levels. - Antioxidant enzyme activity (e.g., CAT, GPx) rose in a time- and dose-dependent manner, suggesting a defense response to ROS. - Gills were more sensitive than digestive glands, indicating higher susceptibility in respiratory tissues. - Chitosan and ionic liquid aggregation reduced NP toxicity in some cases, but effects varied across biomarkers.

PS—polystyrene, PVC—polyvinyl chloride, PS–NH₂—amine-modified polystyrene, IL—interleukin, TNF-α—tumor necrosis factor alpha, cGAS—cyclic GMP-AMP synthase, STING—stimulator of interferon genes, TBK1—TANK-binding kinase 1, IRF3—interferon regulatory factor 3, MDA—malondialdehyde, ROS—reactive oxygen species, SOD—superoxide dismutase, TAC—total antioxidant capacity, LDH—lactate dehydrogenase, H2AX—DNA damage marker, NF-κB—nuclear factor kappa-light-chain-enhancer of activated B cells, Nrf2—nuclear factor erythroid 2–related factor 2, HO-1—heme oxygenase-1, β-hCG—beta-human chorionic gonadotropin, TGF-β—transforming growth factor beta, α-SMA—alpha-smooth muscle actin, Col1a1—collagen type I alpha 1, GPX4—glutathione peroxidase 4, TFRC—transferrin receptor, Fer-1—ferrostatin-1, RAW264.7—mouse macrophage cell line, mtDNA—mitochondrial DNA, BALF—bronchoalveolar lavage fluid, C176—STING pathway inhibitor, ↑—increase, ↓—decrease.

**Table 4:** Particle type and model assessed in each study.

Author and Year	Particle Type	Species	Model/Cells
Busch et al. 2021 [50]	PS, PVC	Human-derived cell lines (*Homo sapiens*)	In vitro triple culture model of the human intestine composed of Caco-2, HT29-MTX-E12, and PMA-differentiated THP-1 cells to simulate healthy and inflamed gut conditions for assessing nanoplastic toxicity.
Huang et al. 2025 [51]	PVC	Humans (*Homo sapiens*)	Human esophageal epithelial cells (HEEC and HET-1A) and human embryonic kidney 293T cells.
Lu et al. 2024 [52]	unspecified	Pig (*Sus scrofa domesticus*)	Two porcine kidney cell lines—IB-RS-2 and PK15.
Poinsignon et al. 2025 [53]	PS	Humans (*Homo sapiens*)	Primary human villous cytotrophoblasts (VCTs) isolated from term placentas.
Weber et al. 2022 [54]	PS, PVC, PMMA	Humans (*Homo sapiens*)	Primary human monocytes and monocyte-derived dendritic cells (moDCs) isolated from peripheral blood mononuclear cells (PBMCs).
Wen et al. 2024 [55]	PS	Mouse (*Mus musculus*)	In vitro triple culture model of the human intestine, consisting of Caco-2 (enterocytes), HT29-MTX-E12 (mucus-producing goblet cells), and PMA-differentiated THP-1 (macrophages).
Ge et al. 2024 [56]	PS	Mouse (*Mus musculus*)	HepG2 human liver cells for in vitro experiments to study polystyrene nanoplastics (PS-NPs)-induced hepatotoxicity and C57BL/6 mice.
Liu et al. 2024 [57]	PS	Mouse (*Mus musculus*)	Mouse-derived RAW264.7 macrophage cells for in vitro experiments and C57BL/6 mice.
Li et al. 2020 [58]	PS-NPs	Asian clam (*Corbicula fluminea*)	Live freshwater bivalves, *Corbicula fluminea*, as an in vivo whole-organism model.
Cid-Samamed et al. 2024 [59]	PS-NH₂ NPs, PS MPs	Mediterranean mussel *(Mytilus galloprovincialis)*	Live Mediterranean mussels (*Mytilus galloprovincialis*) as the in vivo biological model.

Polystyrene (PS); polyvinyl chloride (PVC); polymethyl methacrylate (PMMA); polystyrene nanoplastics (PS-NPs); amine-modified polystyrene nanoplastics (PS-NH₂ NPs) and plain polystyrene microplastics (PS MPs).
